# Consuelo H. Wilkins receives the 2025 ASCI/Louis W. Sullivan, MD, Award

**DOI:** 10.1172/JCI199942

**Published:** 2025-12-15

The American Society for Clinical Investigation (ASCI) honors Consuelo H. Wilkins, MD, MSCI (Figure 1), with the 2025 ASCI/Louis W. Sullivan, MD, Award. Dr. Wilkins is recognized for her paradigm-shifting work identifying health disparities and engaging underrepresented groups in clinical research. Dr. Wilkins is Mildred Thornton Stahlman Chair in Rural Health and Professor of Medicine in the Geriatrics Division at Vanderbilt University Medical Center as well as Associate Director for the Vanderbilt Alzheimer’s Disease Research Center and Engagement Core Director of the All of Us Research Program. She served as Founding Director of the Center for Community Health and Partnerships in the Washington University in St. Louis Institute for Public Health, Codirector of the Center for Community-Engaged Research in the Clinical and Translational Science Awards Program, and Director of Our Community, Our Health. Dr. Wilkins was elected to the National Academy of Medicine and, in 2022, the ASCI. Dr. Sherita H. Golden, Past Chair of the ASCI Physician-Scientist Engagement Committee and Hugh P. McCormick Family Professor of Endocrinology and Metabolism at the Johns Hopkins University School of Medicine, interviewed Dr. Wilkins at the AAP/ASCI/APSA Joint Meeting in Chicago in April 2025.

Sherita H. Golden: As outgoing chair of the ASCI Physician-Scientist Engagement Committee, which reviews candidates for the Louis Sullivan Award, I am delighted to interview our 2025 recipient, Dr. Consuelo Wilkins. Dr. Wilkins, I wanted to ask you what motivated you to focus your research on health disparities and engaging underrepresented groups in clinical research?

Consuelo H. Wilkins: I grew up in a small town in Mississippi, and I witnessed those disparities and inequities in health outcomes very early. It was part of what motivated me to be a physician. I saw people that I loved suffer from poor health; they didn’t have great access to healthcare; [they had] a small hospital that eventually closed. And it led me to see how much care was needed in some communities. I decided to go into medicine early on, but it wasn’t until a residency, when I was at Duke and seeing how the evidence that had been generated wasn’t applicable to everyone, that I was pushed more in the direction of science and thinking through what evidence needs to be [built] and how we need to make sure that all the populations that we care about, especially those experiencing disparities and inequities, are included in clinical research.

SHG: What factors do you feel are most critical in your research success?

CHW: Obviously there are things like having good training, having amazing mentors, understanding how to do good, rigorous science, the skills, the methods — all of those things are incredibly important. For me, community was important. Community engagement, learning and understanding the differences among communities, was eye-opening. When I was early in my career, I was on the faculty at Washington University, and one of my first studies was focused on Alzheimer’s disease and cognitive impairment, and I wanted to look at some of the disparities. Black, African American, Latinx people are more likely to have Alzheimer’s. I needed to engage more specifically Black men, as we did not have enough Black men in the study. I’m going out in the community; I have connections. I get connected with retired police and firefighters in St. Louis. It’s predominantly a group of Black men. I’m telling them about how great this project is and why they should be engaged. And they responded, “Hmm . . . Interesting . . . ” I was talking about mortality, using all the sophisticated language and data, and they basically said, “Well, we’re all going to die. We’re not afraid.” This group of men, especially in their professions, they’re not afraid of dying. I’m telling them about death, and they ask, “What else have you got?” They want to know why they should participate, how this is going to improve their quality of life, give them more time with their families. It should have been obvious that these are things that would be important. But they made it clear that if I wasn’t thinking about what they needed and how this would impact their lives, what could be important to them, they had no interest. That really changed my ideas and the way I approached science.

SHG: That community engagement is so critical to the success of any research.

CHW: And of course, we didn’t learn about it in any of the training. It’s not in my master’s in science clinical investigation program. No community engagement, period.

SHG: Fortunately now there’s a field of community-based participatory research that teaches those skills. But when we both trained, that would not have been the case.

CHW: At least not for physicians.

SHG: Were there obstacles to getting where you are today?

CHW: So many challenges, and I think specifically from the science piece early in my career, the idea of doing research in disparities and inequities was not as valued. It was presumed that “It’s good, important work, but it’s not necessarily science.” I was pushed to do more mechanistic work and to think about some of the more cutting-edge technologies and radiology, especially around Alzheimer’s, and about imaging. I was told, “We love that you’re interested in this, but that’s not going to get you the publications and the high-impact journals. It’s not going to get you funded.” So I was really discouraged from pursuing this as a scientific career, [as opposed to] something I should just do on the side. I think that the whole idea that this work was devalued created so many challenges — again, to the funding, to the kinds of support, to me having parallel projects happening: I’m going to do this project that’s looking at neuroimaging because that’s how I can get funding, but I’m going to do this work on the side because that’s where I’m really interested. But of course, now we have had, at least until recently, so many more opportunities for funding in health disparities, health inequities, as well as community engagement. Other barriers to the work are understanding the methodology — again, a lot of that has been built in the last couple of decades — but having training and access to resources to support the science, those were certainly challenges early on. We could spend a lot of time talking about discrimination and racism and other things that I experienced. But I don’t know that that’s different from [the experience of] many other people who are from marginalized groups. This is not to normalize it, but [to emphasize a situation] unique to my specific area.

SHG: Where do you see the work going in the coming years? And I know you have a leadership role at Vanderbilt working in this space.

CHW: I think we’re at an important point to think about how we better communicate about our work and health disparities. In the last few months, we’ve seen the language “health disparities” and “health inequities” be villainized and devalued in different ways. I think this is where we need to double down on the support for this kind of work and research. We continue to see disparities and inequities that impact everyone. The idea that only those communities that are directly impacted suffer is incorrect. We all suffer from seeing some people have worse health outcomes. I think that there are going to be even more challenges to doing the work; having to describe it in terms that essentially whitewash it, take away the meaning, is going to make it more challenging to communicate. But we still have many opportunities to focus on the needs of the population, the disparities and inequities. This is where we really have to, as scientists, lean into the data. I think we are not doing enough pushing back with the evidence that these are not ideologies. These are facts that outcomes are worse. It’s poor science — obviously you study diabetes — if you are doing a study of diabetes and you don’t have the people who are most impacted by diabetes in the study, it is not good science. For so long, we have allowed this kind of research to happen where we’ve not actually included the full range of people who have conditions. I think we need to start rejecting that, to stand up more in all areas in the academy, in our respective institutions, in publishing, in the advocacy organizations. This is where we need to really lean into: What do we need to maintain the infrastructure and support and advance this work and more effectively communicate about it?

SHG: Because effective communication is important, because the disparities that impact health aren’t limited to racial demographic groups but [affect] multiple demographic groups: geography, rurality, disability.

CHW: And I mentioned I grew up in a small town. I didn’t consider it to be rural: that was all I knew at the time. My town had two stoplights, so we were big! But there are lots of small communities around it. I was recently awarded an endowed chair focused on rural health, the Mildred Thornton Stahlman Chair in Rural Health, and I’m looking forward to using that as an opportunity to show how these inequities and disparities are apparent across many communities, but also how over time we’ve actually continued to silo and marginalize people by not recognizing the similarities in some of these disparities, the social and structural drivers. They may be slightly different, but a lot of them are common.

SHG: What lessons have you learned in your own training, and what advice do you have for trainees or young physician-scientists?

CHW: First, I would say that I’m so inspired by the young, early-career physician-scientists. They are amazing. They are thinking more broadly about work and how to do it and about the populations that are impacted. That certainly gives me joy that they are willing to stand up and ask the right questions, in addition to realizing that you need to understand the communities that are impacted and demonstrate trustworthiness and be able to actually connect with them. That’s important in terms of the skill sets you need to be a good physician-scientist — and effectively communicating the cultural humility that [is needed] to do that. When we’re writing our med school applications, we’re saying things like “We want to help everybody. We want to communicate; we want to save the world.” In some ways in medical school and in training, we try to strip all those things out in different ways: “Just be the expert, and don’t care about people as much.” I’m sure it’s not consciously that we’re doing that, but we need to ensure that [trainees maintain and actually strengthen that attitude]. So learning how to engage communities is important. If you’re involved in health disparities, health inequities [research], you need to understand the social, structural factors. Often that means you need to partner with a social scientist if you’re not a social scientist. We tend to bring those people in at the end, when we’ve already developed the protocols and written the grants, and then we just want them to make it better or recruit people for us. We are missing a key piece of that: you need a social or structural determinants of health conceptual framework to lead the work. I would say, again back to the communication: It doesn’t matter if it’s at the cellular level, if it’s basic science research: wherever you are on the spectrum, you need to be able to clearly communicate. We’re seeing that is missing right now as people are confused about what we’re doing, conflating terms. And this is an important opportunity to learn and make sure we know how to tell the stories behind the work. The last thing I would say for young physician-scientists is to be bold and curious with your questions. Don’t be afraid to ask us why we didn’t do something or why we did something one way. Again, the questions, the way that the students have pushed us in thinking about things like race-based algorithms and things that we have accepted and need to deimplement — I think we need more of that, and we should be encouraging them to do that.

SHG: It has really been a delight to interview you. Thank you for your time.

CHW: My pleasure.

*The interview has been edited for length and clarity*.

## Figures and Tables

**Figure 1:**
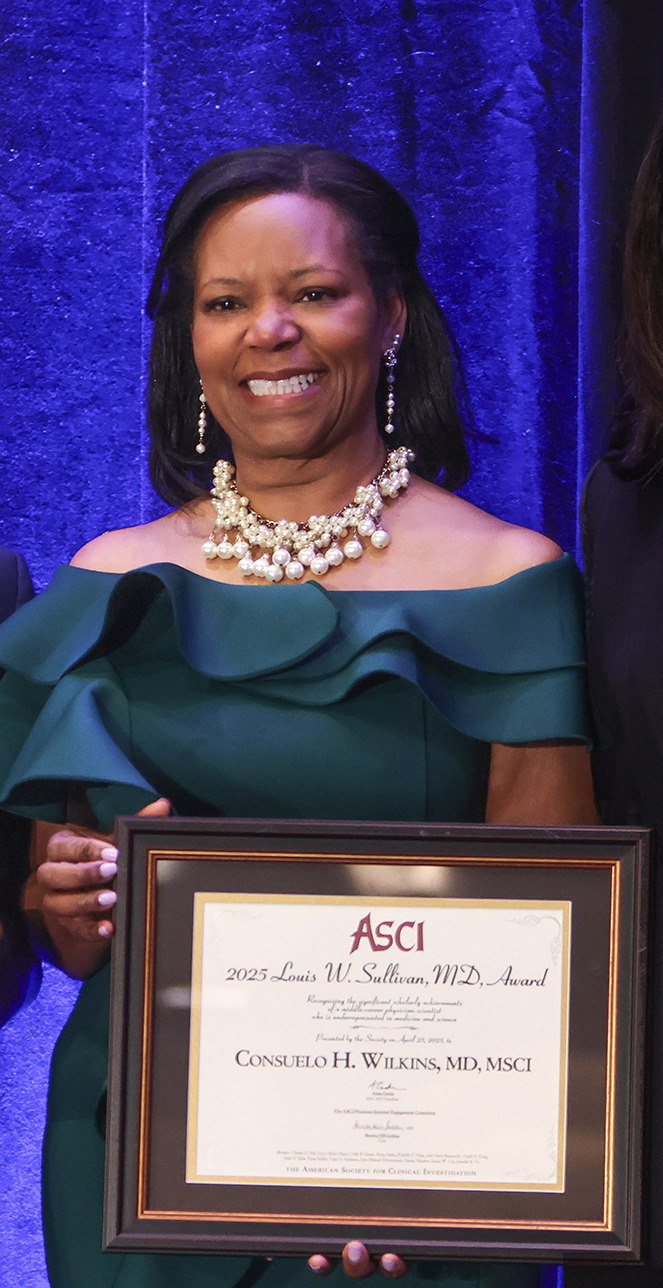
Consuelo H. Wilkins is the recipient of the 2025 Louis W. Sullivan, MD, Award.

## Version 1 12/15/2025

Electronic publication

